# Personalizing digital pain management with adapted machine learning approach

**DOI:** 10.1097/PR9.0000000000001065

**Published:** 2023-02-13

**Authors:** Yifat Fundoiano-Hershcovitz, Keren Pollak, Pavel Goldstein

**Affiliations:** a Dario Health, Caesarea, Israel; b Integrative Pain Laboratory (iPainLab), School of Public Health, University of Haifa, Israel

**Keywords:** Posture biofeedback, Back pain, Digital therapeutics, Machine learning, Decision tree, Mixed model, Personalized digital therapeutics

## Abstract

**Introduction:**

Digital therapeutics (DT) emerged and has been expanding rapidly for pain management. However, the efficacy of such approaches demonstrates substantial heterogeneity. Machine learning (ML) approaches provide a great opportunity for personalizing the efficacy of DT. However, the ML model accuracy is mainly associated with reduced clinical interpretability. Moreover, classical ML models are not adapted for the longitudinal nature of the DT follow-up data, which may also include nonlinear fluctuations.

**Objectives:**

This study presents an analytical framework for personalized pain management using piecewise mixed-effects model trees, considering the data dependencies, nonlinear trajectories, and boosting model interpretability.

**Methods:**

We demonstrated the implementation of the model with posture biofeedback training data of 3610 users collected during 8 weeks. The users reported their pain levels and posture quality. We developed personalized models for nonlinear time-related fluctuations of pain levels, posture quality, and weekly training duration using age, gender, and body mass index as potential moderating factors.

**Results:**

Pain levels and posture quality demonstrated strong improvement during the first 3 weeks of the training, followed by a sustained pattern. The age of the users moderated the time fluctuations in pain levels, whereas age and gender interactively moderated the trajectories in the posture quality. Train duration increased during the first 3 weeks only for older users, whereas all the users decreased the training duration during the next 5 weeks.

**Conclusions:**

This analytical framework offers an opportunity for investigating the personalized efficacy of digital therapeutics for pain management, taking into account users' characteristics and boosting interpretability and can benefit from including more users' characteristics.

## Abstract 2

The presented analytical framework offers an opportunity to investigate digital therapeutics' personalized efficacy for pain management, considering users' personal characteristics and boosting model interpretability.

## 1. Introduction

During the last decade, with health care becoming increasingly digital, digital therapeutics emerged and has been expanding rapidly. Relying on various digital platforms, digital therapeutics' goals are to change patient behavior and assist with treating medical conditions. Various digital therapeutic approaches have shown promising clinical efficacy in managing chronic diseases, smoking, addictions, sleep conditions, obesity, cardiovascular diseases, and chronic pain^8^. The therapeutic effects are usually administered in a population-based and one-size-fits-all fashion,^27^ whereas the treatment efficacy generally differ between patients. However, digital data provide an excellent opportunity to use machine learning (ML) algorithms to uncover personalized treatment efficacy based on patient characteristics. Such algorithms may vary in their accuracy and clinical interpretability. Although these 2 criteria mostly show reverse dependency, more accurate ML approaches are associated with lower interpretability. Therefore, significant effort has been focused on balancing the accuracy and interpretability of ML approaches.^16^

Multilayer neural networks that model complex data patterns predicting clinical outcomes are called deep learning. As deep learning has received remarkable attention for its capacity to achieve accurate predictions, there is a growing understanding that better interpretability of these ML methods is required. On the other side of the scale, classical regression-based approaches are associated with low model accuracy but more explicit model interpretation.^3^ Since both model accuracy and interpretability are highly required, a tradeoff solution is needed.

Decision trees (DTs) look like an upside-down tree that starts with a root node with outgoing branches that feed into the internal nodes, also known as decision nodes. The process of dividing a single node into multiple nodes is called splitting. If a node does not split into further nodes, then it is called a leaf node or terminal node. A subsection of a decision tree is called a branch or subtree. DTs can model nonlinear effects and are easily interpretable if the tree depth is reasonable.^16^ Decision Trees result in a reasonable tradeoff between model accuracy and interpretability, providing clinicians and patients with a substantial opportunity to reach the best decision, gaining the trust of the treating clinician.^11^ Decision trees is a simple, easily interpretable approach with clinically valued solutions. Although other ML approaches may result in higher accuracy, DTs was selected due to the clear explainability of the whole model with interactive effects. Such a framework sheds light on the mechanisms underlying the model, providing important clinical insights in addition to the predictive value.

Despite the potential of ML to improve the efficiency of digital therapeutics, there is still a challenge in adapting and implementing ML. This is partially related to most digital therapeutic data's complexity and longitudinal nature. Classical ML algorithms assume that the training data are independent and identically distributed. However, this assumption is frequently violated in real-world follow-up data, primarily representing patients' fluctuations over time, highlighting the need to account for interpatient and intrapatient variability. Moreover, intrapatient fluctuations may have nonlinear patterns, challenging the modeling process.

To overcome these issues, we suggest using an integrative approach that combines implementation of DTs with a framework of piecewise mixed models for modeling nonlinear follow-up trajectories. First, a model can be fitted for modeling time-related trajectory for the general population. Then, the DTs algorithm can be applied to personalize the model for the learned subgroups of patients based on their characteristics.

Digital therapeutics approaches have been successfully used for treating low back pain.^4^ Low back pain is the leading cause of years lost because of disability and has a reported lifetime prevalence of up to 84%.^1^ Although posture biofeedback training has demonstrated promising efficacy in pain management,^7,15^ we still lack a deep understanding of the biofeedback's temporal dynamic and factors affecting the efficacy. In this follow-up study, we developed predictive models for weekly training duration, pain levels, and posture quality using piecewise mixed-effects model trees considering the data dependencies and time-related nonlinear trajectories. Although the clinical value of the findings, here, we focus on providing hands-on experience in the implementation of analytical tools for personalized pain management using adapted ML techniques.

## 2. Methods

### 2.1. Platform

This study used a postural biofeedback feedback wearable device (UpRight by DarioHealth) to collect follow-up data between 2018 and 2020. The device is programmed to vibrate when slouching postures are detected (based on the change in tilt and curvature of the spine) and alert users of their change in body position. Visual feedback is also available through the cell phone screen. The device is removed from its holder, placed in the middle upper back under the shirt, and directly connected by Bluetooth to a cell phone, effectively converting the cell phone into the display screen for the device. The UpRight device is a postural training device that uses a triaxial accelerometer to set target posture and monitor body position to provide a vibration stimulus alert when optimal body posture is not being maintained. Connecting the device directly to the phone ensures real-time data capture during a posture training session. In a “Calibration” screen, the user calibrates upright to their back. The user is instructed to sit upright and stay still once ready to press the “I'm upright” button. The device records the upright position of the user.

### 2.2. Measures

Training duration (the time the device is worn) was recorded. After each session, users were asked to rate progress by logging back the pain level using a numerical rating scale on a 0 to 10 scale^15^ (0–no pain; 10–extreme pain) and posture on a 0 to 10 scale (0–mostly slouched; 10–mostly upright) in data entry screens. The weekly average pain and posture levels were used as core outcome metrics. Total training duration was calculated for the same 7-day intervals. In addition, users' demographic variables such as gender, weight, height, and age were collected. All data were transferred and stored in PostgreSQL and BigQuery database services. All data were anonymized before extraction for this study.

### 2.3. Users

UpRight platform users were screened with the following inclusion criteria: high pain levels (>4) at the first assessment, at least 2 ratings in the system, and at least 8 weeks of training sessions of above 6 hours (weekly). A group of 3610 users was included in this analysis—59.66% women with average age 43.66 (SD = 16.05) and average BMI 23.94 (SD = 5.67) and 40.33% men with average age 44.06 (SD = 15.33) and average body mass index (BMI) 25.42 (SD = 3.71) as presented in Table 1. Since not all users used self-reporting assessments, the data set of 2479 participants was used for modeling pain levels and posture quality.

**Table 1:** Descriptive statistics of the sample.

Characteristic	Overall, N = 3,610*	By gender		*P* ^†^
		Female, N = 2,154	Male, N = 1,456	
BMI				<0.001
N	3,610.00	2,154.00	1,456.00	
Median (IQR)	23.89 (21.55, 26.54)	22.77 (20.70, 25.86)	25.00 (23.15, 27.17)	
Range	11.07, 103.05	11.07, 103.05	14.36, 53.15	
Mean (±SD)	24.54 (±5.03)	23.94 (±5.67)	25.42 (±3.71)	
Age (y)				0.12
N	3,610.00	2,154.00	1,456.00	
Median (IQR)	40.00 (32.00, 54.00)	40.00 (31.00, 56.00)	41.00 (32.75, 52.00)	
Range	15.00, 94.00	15.00, 93.00	15.00, 94.00	
Mean (±SD)	43.82 (±15.76)	43.66 (±16.05)	44.06 (±15.33)	

* c(“N,” “median (IQR),” “range”, “mean (±SD)”).

† Wilcoxon rank-sum test. BMI, body mass index.

Ethical & Independent Review Services,^10^ an AAHRPP accredited, service-oriented company that provides Institutional Review Board review of human subjects research, issued the institutional review board exemption for this study (21048-01#).

### 2.4. Study design

The data analysis was performed using R software.^21^ As count data, biofeedback weekly training duration naturally has right-shifted distribution; therefore, it was log-transformed to better fit the model assumptions. First, linear mixed-effects models (LMMs) were used to define the time-related fluctuation for pain levels, posture quality, and weekly biofeedback training duration over 8 weeks. LMMs provide a statistical framework for modeling repeated measurements by taking into account the different types of data dependencies.^2^ The piecewise approach allows the modeling of multiple linear trends over time, providing an opportunity to model curvilinear changes in the weekly pain level, posture quality, and weekly training duration as an integral process.^14^

**Figure 1.**
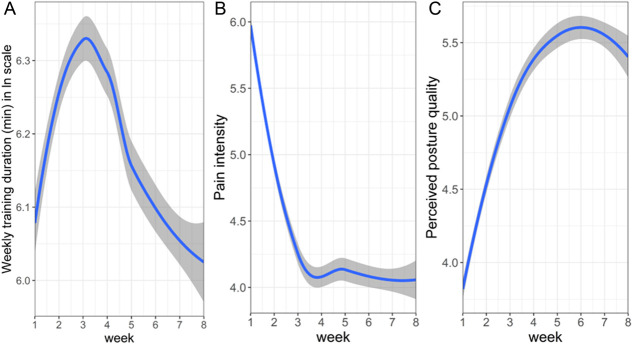
Visual presentation of the outcomes over 8 weeks. (A) Log-transformed biofeedback weekly training duration; (B) pain intensity; (C) posture quality. The blue lines show LOESS smoothing, with grey areas representing the corresponding 95% confidence intervals.

For the current study, 2 linear trajectories (piecewise components) were integrated into a mixed model, assuming break points at weeks 3 and 4 based on previous studies and the data visualization (Fig. 1).^14^ Model fit was evaluated by Akaike information criterion (AIC), and the most suitable models were selected for further analysis. Parameterization and implementation of a piecewise mixed model for longitudinal data were explicitly demonstrated by Magnusson.^17^ Next, the best model for the general population from the previous stage was used for LMM tree analysis. The LMM tree algorithm learns a tree where each terminal node is associated with different fixed-effects regression coefficients while adjusting for global random effects (random intercept or random slope). This allows the detection of users' subgroups with varying fixed-effects parameter estimates, keeping the random effects constant throughout the tree.^12,13^ As mentioned, the LMM tree algorithm was incorporated with piecewise parameters to detect subgroups with different piecewise trajectories according to the potential moderating factors of age, gender, and BMI. This approach may assist in identifying a specific nonlinear trajectory for the subpopulations characterized by the moderating factors.

Unfortunately, applying ML on a single data set may result in overfitting. Overfitting is a condition that occurs when a ML model that shows a good fit for a specific data set then cannot be generalized to a new data. Therefore, the data set was randomly partitioned (by users) into 80% train set,which will actually be used to adjust the parameters of the models, and 20% test set—which will be used to measure the predictive validity of the chosen model during the training. All LMM tree models were built using the lmertree function from R package “glmertree”^13,21^ under the assumption of random intercept for the users, meaning that the model considers that there is a difference in outcome between individuals. In addition, random slope for the first piecewise component, which stands for the initial training period, was assumed, meaning that the outcome trajectory (pain, eg,) along the first 3 weeks might be different for each person, or in other words, the progress of treatment might differ from one person to another. Thus, the implementation of lmertree function in general looks as follows:

lmertree(outcome ∼ picewise_parameter1 + picewise_parameter2 | user ID | potential partitioning variables,…pruning hyperparameters, data = your_data)

Pruning is a critical step in applying a decision tree model. Pruning is commonly used to alleviate the overfitting issue in decision trees. The prepruning process was deployed as all models held “minsplit” (the smallest number of observations in the parent node that could be split further) parameter constant 90 for the pain levels and posture quality (at 100 the model fail to converge due to model complexity) and 100 for the “weekly training duration” and while “maxdepth” the maximum depth of the tree, in other words, the maximum distance between the root and any leaf) parameter was evaluated in a range of 2 till 5 (to reduce overfitting). Therefore, the final model which was deployed incorporates also the “maxdepth” and “minsplit” parameters:

lmertree(pain ∼ w1_3+w3_8 | (1 + w1_3 | id) | bmi+age+gender, data = our_train_data, maxdepth = 5 , minsplit = 90)

The most suitable models were selected by assessing AIC.

Five-fold cross-validation for pain levels and posture quality were deployed on the training data. Finally, the models were tested with an untouched (test) data set, whereas the assessment of the model's performances was evaluated with root mean square error (RMSE), comparing RMSE based on the training and test data.

## 3. Results

Visual presentation of the weekly training duration, pain duration, and posture quality over 8 weeks appears in Figure 1.

Based on Figure 1, a piecewise mixed model was fitted with a cut-off point at 3 weeks and 4 weeks to define the best model that describes the fluctuation of the 3 outcomes over time. In addition, a model with a single linear slope was fitted. The piecewise model with a cut point at week 3 showed the best fit based on AIC for all 3 outcomes. For this reason, LMM trees were applied with piecewise 2-slope parameters using a cut point at week 3. The piecewise mixed-effects model trees were trained for all 3 outcomes (weekly training duration, pain levels, and posture quality) to find the best group separation for the piecewise time trajectories based on the predefined set of potential moderators—age, gender, and BMI.

For example, the code for the pain model can be presented as:

lmertree(pain ∼ w1_3+w3_8 | (1 + w1_3 | id) | bmi + age + gender, data = our_train_data)

Where:

(w1_3 + w3_8)—piecewise parameters for weeks 1 to 3 and 3 to 8 correspondently.

(1 + w1_3 | id)—random intercept and random slope for the weeks 1 to 3.

BMI + age + gender – moderators\partitioning variables.

### 3.1. Biofeedback weekly training duration

**Figure 2.**
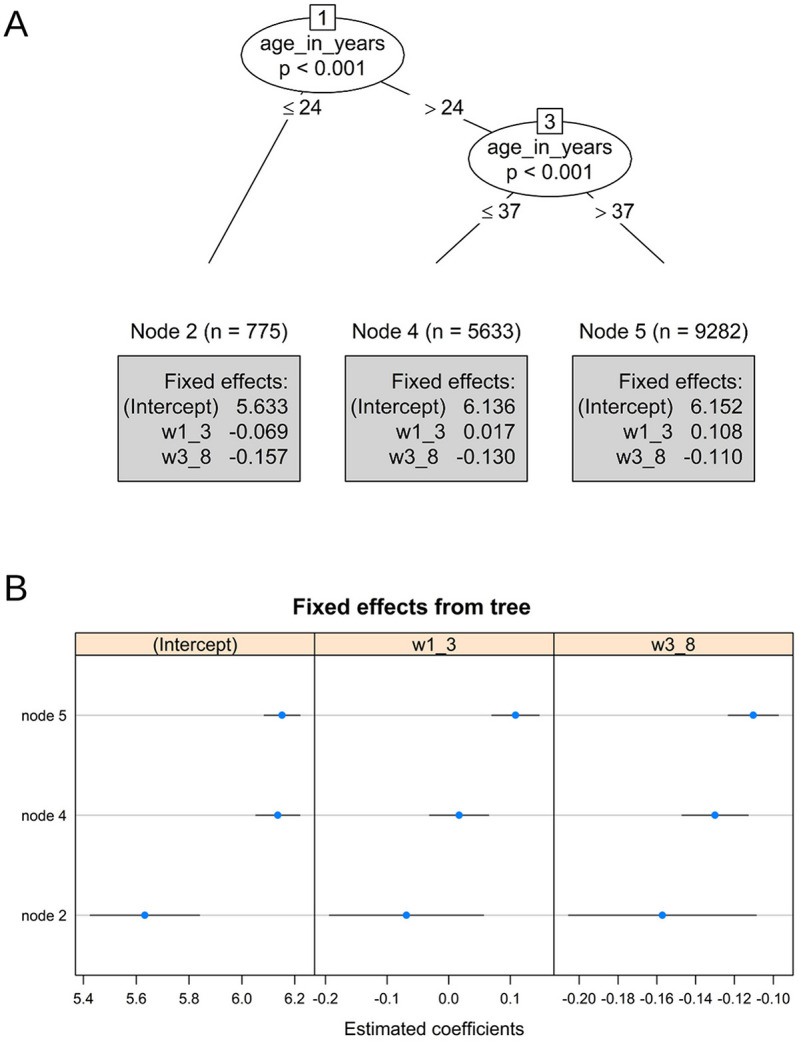
(A) Piecewise mixed-effects model tree with estimated coefficients printed in the terminal nodes for the biofeedback weekly training duration. w1_3 represents the estimate of the first time-related slope (weeks 1–3), and w3_8 represents the estimate of the second slope (weeks 3–8). (B) Caterpillar plot of estimated node-specific fixed effects with 95% confidence intervals for the biofeedback weekly training duration.

Figure 2A presents the model with estimated coefficients of biofeedback weekly training duration. As can be seen, the model resulted in 3 subgroups, based on age as the meaningful moderating factor.

Since the training duration was log-transformed, the exponential transformation was used for the back transformation to the origin units. The first group, which represents the younger individuals (node 2, age lower than 24), tended to practice for ∼279.5 minutes at the beginning point. The second (node 4: 24 < age ≤37) tended to practice 462.2 minutes, whereas the third group, which represents the older individuals (node 5, age greater than 37), was practicing for 469.66 minutes on average. As can be seen, younger individuals tend to practice less (Fig. 2).

Regarding the first slope (W1_3, which represents the 3 first weeks), only node 5 shows a significant increase (*P* < 0.001) in training duration for ages older than 37. Hence, we can conclude that for older individuals, during the first 3 weeks, the total practice duration rises by 11.4% [(EXP(0.108) − 1) × 100)]. The other 2 groups, the young (age ≤24) and the midage (24 < age ≤37), remain stable, and we cannot specify any trend (*P* = 0.144 and *P* = 0.294, respectively).

The second slope (W3_8: weeks 3–8) showed significant training reduction across all age groups. However, the pace of the reduction increased with age. For node 2 (age ≤24), node 4 (24 < age ≤37), and node 5 (age >37), the weekly training duration decreased by 14.52% (*P*-value <0.001), 12.19% (*P*-value <0.001), and 10.42% (*P*-value <0.001), correspondingly.

### 3.2. Pain levels

**Figure 3.**
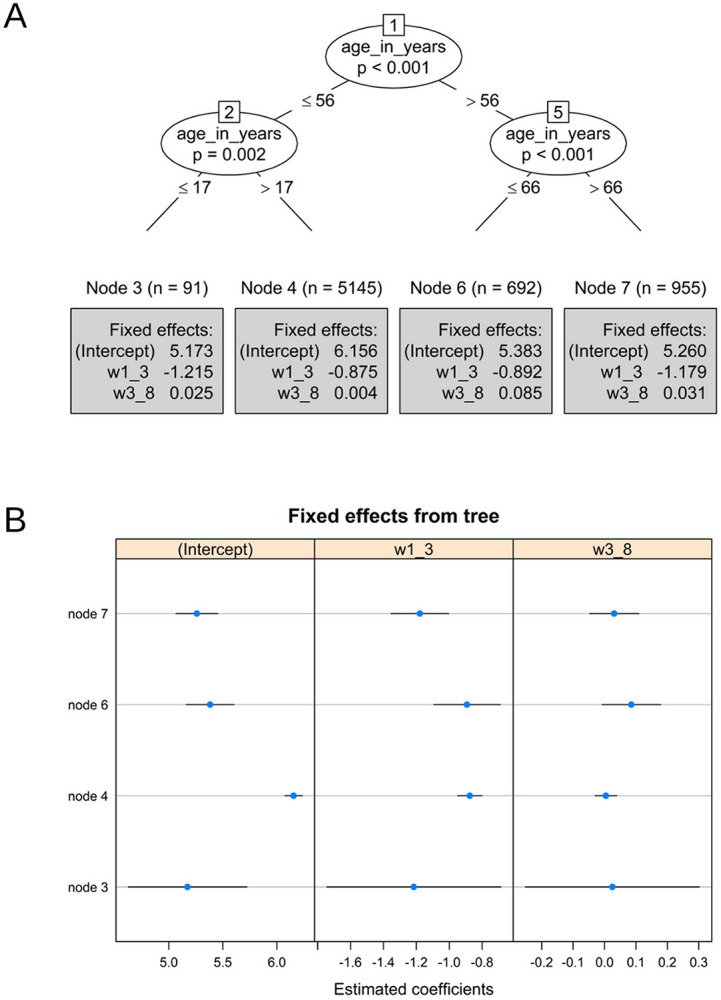
(A) Piecewise mixed-effects model tree with estimated coefficients printed in the terminal nodes for pain levels. w1_3 represents the first time-related slope (weeks 1–3). w3_8 represents the second slope (weeks 3–8). (B) Caterpillar plot of estimated node-specific fixed effects with 95% confidence intervals for pain levels.

Figure 3 presents the model for predicting pain levels. The model defined 4 subgroups based on age as the meaningful moderator: node 3—the younger individuals (age ≤17); node 4 to 17 < age ≤ 56; node 6 to 56 < age ≤ 66, and node 7—older individuals (age > 66).

Based on the model intercepts, all groups (except Node 4: 17 < age ≤ 56) start at similar pain levels (5.173, 5.383, and 5.260), whereas the group of 17 < age ≤ 56 has higher pain levels (6.156).

Based on Figure 3, all 4 groups experienced a significant reduction in pain levels over time during the first 3 weeks (*P* < 0.001 for all). Interestingly, the 2 “utmost” groups (age ≤17 and age >66) were characterized by the greatest pain reduction.

Regarding the trends in the second period, the group defined by 56 < age ≤ 66 showed a increase in pain levels (*P* = 0.02). However, the pain increase rate was minor compared with the initial pain reduction. All other age groups showed a stable pain level pattern over the second period (node 3: *P* = 0.825; node 4: *P* = 0.751; node 7: *P* = 0.306).

### 3.3. Posture quality

**Figure 4.**
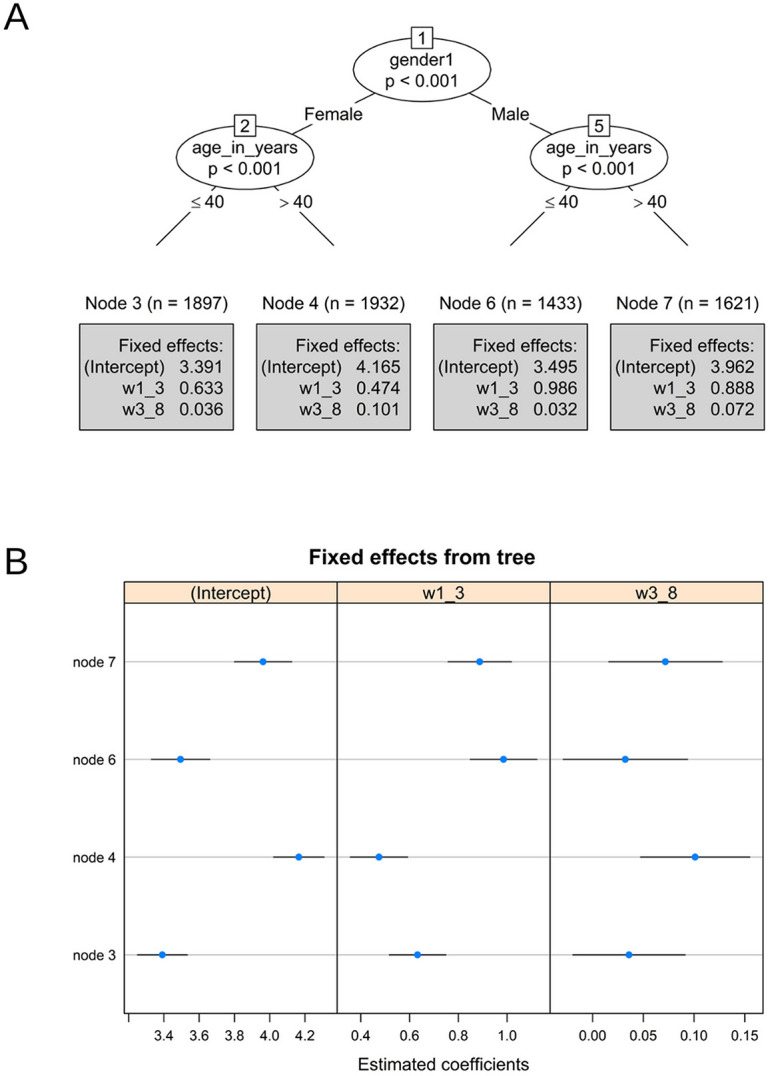
(A) Piecewise mixed-effects model tree with estimated coefficients printed in the terminal nodes for posture level. w1_3 represents the first time-related slope (weeks 1–3). w3_8 represents the second slope (weeks 3–8). (B) Caterpillar plots of estimated node-specific fixed effects with 95 confidence intervals for posture quality.

Figure 4 shows the findings for the posture quality model, which consists of 4 subgroups based on gender and age as the meaningful moderators: node 3: women at age ≤40, node 4: women at age >40, node 6: men at age ≤40, and node 7: men >40.

Initial posture quality is similar across all 4 identified subgroups (Fig. 4A). Women and men below age 40 show similar posture quality (3.39 and 3.50, respectively), whereas women and men over 40 showed increased posture quality (4.17 and 3.96, respectively). All 4 subgroups significantly increased posture quality over the first 3 weeks. Men in both age groups (both *P*'s < 0.001) showed a stronger posture improvement relative to women (both *P*'s < 0.001).

For the second period, women and men 40 and younger demonstrated stable posture quality (*P* = 0.08 and *P* = 0.13, respectively), whereas women and men older than 40 years of age showed increased posture quality (both *P*'s < 0.001).

Table 2 represents averaged RMSE values, comparing the model fit for the cross-validated training and test data. As expected, test data show a slight increase in RMSE.

**Table 2:** Model's performance based on RMSE.

Outcome	RMSE—training	RMSE—test
Training duration	1.100091	1.40267
Pain	1.835511	2.19211
Posture	1.735765	2.097656

RMSE, root mean square error.

## 4. Discussion

### 4.1. Principal findings

This study demonstrated the implementation of piecewise mixed-effects model trees for predicting nonlinear temporal fluctuations of posture biofeedback training duration, associated pain levels, and posture quality of the users incorporating personal characteristics as moderating factors. Age moderated the time-related trajectories of biofeedback training duration and pain, whereas age and gender moderated the trajectories of posture quality. Specifically, although all users started from a comparable level of training duration, the relatively older users (older than age 37) significantly increased their training over the first 3 weeks compared with the younger group (younger than age 37), which remained at stable training levels over the following 5 weeks. A group of users younger than age 24 maintained a steady level of training in their first 3 weeks and did not increase. In the second trajectory from weeks 3 to 8, all 3 groups showed significant training reduction, whereas the younger users demonstrated a steeper reduction in the training duration.

The 4 groups significantly reduced pain levels during the first 3 weeks. Remarkably, the 2 “utmost groups” (age ≤17 and age>66) were observed with the greatest pain reduction. Three age groups showed stable reduced pain except for the group at 56 < age ≤ 66, which showed a minor increase in pain levels. By contrast, age and gender moderated time-related fluctuations in posture quality. All users significantly improved their posture during the first 3 weeks. However, men showed stronger posture improvement than women. During weeks 3 to 8, women and men at age ≤40 remained stable in their posture quality, whereas women and men older than age 40 demonstrated an additional increase in posture quality.

Traditional health care has limited resources for meeting all patients' needs, especially providing nonbiomedical solutions. Digital therapeutics can deliver evidence-based therapeutic interventions that are driven by digital platforms and connected devices to prevent or manage a medical condition or disease.^20^ However, recent studies reported strong heterogeneity in the efficacy of various digital therapeutic approaches that may be systematic and rely on patients' personal characteristics. Understanding trends and progress in different populations (ie, by age groups and gender) can be used to support therapy or personalized educative content to optimize user care and health outcomes.

Interestingly, this study showed that relatively older users (older than age 37) significantly increased their training duration over the first 3 weeks compared with the younger group. Of 134.5 million adults in the United States who reported MSK conditions, older adults experience higher prevalence rates of MSK conditions and limitations than younger adults.^18^ Past research indicated that older generations used a digital MSK program more than younger generations showing how older adults who completed digital programs exceeded the completion rates of younger generations.^26^ Older generations also show increased digital engagement, spending more time on exercising, reading educational materials, and communication with coaches, compared with younger adults.^26^ One of the reasons for increased usage of digital programs in older adults is probably the link between age and the attitude about digital health technology for self-management. First, paying more attention to MSK pain, the older population may be more likely than younger adults to use the digital MSK program.^28^ Second, users of older generations may have appreciated that the programs enabled them to manage their needs themselves and at home.^9^

In addition, our findings show that women and men older than age 40 demonstrated a relative increase in posture quality during weeks 3 to 8 of training following the improvement during weeks 1 to 3. Additional improvement in posture perception in the second period may be explained by the fact that using upright and positively increasing posture awareness might encourage cognitive–behavioral modes for pleasant experiences or valency.^6^

Moreover, older users (older than age 66) demonstrated more substantial pain reduction and stronger digital engagement than other age groups. Posture biofeedback, as a part of digital therapeutics, focuses on providing frequent treatment to patients to improve their behavior and lifestyle habits.^22^ Posture biofeedback training has been previously shown to reduce pain and increase physical activity.^19^ Specifically, a previous study showed more substantial pain reduction associated with posture biofeedback in older adults.^4^

We have also observed that men showed a higher increase in self-reported posture improvement than women. Previous research performed on gender differences in body estimation supported that men are less dissatisfied with their own body and consider themselves to be better looking.^25^ Various factors have been discussed as potentially contributing to the differences in body image between women and men, one of them may be due to the fact that women are more frequently challenged with ideal bodies in the media than men.^5^

Our findings provide valuable insights about the nonlinear efficacy of digital therapeutic platforms that varies based on users' characteristics. Machine learning approaches should have more robust applications, analyzing user-based trajectories of digital therapeutics, especially modeling nonlinear time-related fluctuations. Our approach identifies relevant moderating factors of digital follow-up data, taking advantage of data-driven strategies and shedding light on personalized therapeutic effects. Here, we used a decision tree approach integrated with piecewise mixed models, providing a framework for modeling nonlinear trajectories of longitudinal user trends during the biofeedback training. This framework offers an opportunity to boost the clinical interpretability of the findings, highlighted as a current goal of ML evolution. However, other ML approaches can be potentially used based on the task.

Similar computational approaches could be also used with python library gpboost, applying GPBoost algorithm.^23,24^ GPBoost combine gradient tree boosting along with mixed effects models, enabling nonlinear effects (eg, piecewise slopes). GPBoost is different from R package glmertrees, because it uses a DTs boosting algorithm that usually combines set of decision trees, whereas glmertrees suggests using generalized linear mixed-model tree algorithm which based on model-based recursive partitioning, which generates only a single decision tree, characterized with more clinically, intuitive interpretation for results, relatively to GPBoost.

In this study, our analytical approach identified a more substantial efficacy of the posture biofeedback for older people, raising questions about the need to modify training programs for younger adults. Moreover, this approach can be used to assess whether other developed features or programs demonstrate efficacy during product usage. The rationale is to spot data structures that correspond with a known preclassification, such as the presence of a moderating factor of the training and training outcomes' phenotypes. This concept is also cored to personalized medicine, which posits that since individual users have unique characteristics at social–demographic, physiological, and behavior levels, they may need to have adapted interventions tailored to these nuanced and unique characteristics.

### 4.2. Limitations

We noted several limitations in this study. First, as in most retrospective follow-up studies with digital therapeutics tools, a control group was not implemented, reducing the validity of the inferences. Also, other personal characteristics (not collected here) might explain or improve the model accuracy.

As a real-world data analysis, the timescale was designed to reflect the weekly interval change over the 3 weeks and the 3 to 8 weeks period after starting the use the digital biofeedback device. However, the biofeedback efficacy could be potentially evaluated on different scales emphasizing daily or monthly fluctuations. Owing to the difficulty in tracking daily changes in real-world studies, most studies focus on weekly fluctuations. Other ML approaches could be applied to compare the model accuracy and interpretability. Finally, here we use a random sample split approach for the model validation. However, given the nature of ML being optimized for specific data sets, the model generalizability can only be shown by validating the model with data collected by other teams in various geographical areas.

## 5. Conclusion

In this study, we provide an analytical framework that could be used for investigating the personalized long-term efficacy of digital therapeutics for pain management, taking into account various users' characteristics. This framework can be easily applied for testing the personalized efficacy of other therapeutic approaches and clinical cases. Also, our approach can be adopted for comparing various multisession therapies, considering the heterogeneity of the efficacy for various subgroups of the users.

## Disclosures

Y. Fundoiano-Hershcovitz is an employee of DarioHealth, the company that manufactures the upright device and platform; P. Goldstein has received a consulting fee to assist with analyses, but otherwise has no conflicts of interest. The Israel Data Science Initiative (IDSI) of the Council for Higher Education in Israel and the Data Science Research Center at the University of Haifa supported the study.
